# Variation in effectiveness of the NHS Diabetes Prevention Programme in people diagnosed with non‐diabetic hyperglycaemia by age, sex, BMI, and deprivation: A matched cohort analysis of 69,801 people

**DOI:** 10.1111/dme.70037

**Published:** 2025-04-18

**Authors:** Rathi Ravindrarajah, Matt Sutton, Peter Bower, Evangelos Kontopantelis

**Affiliations:** ^1^ Division of Nursing, Midwifery and Social Work, Faculty of Biology, Medicine and Health University of Manchester Manchester UK; ^2^ NIHR School for Primary Care Research, Faculty of Biology, Medicine and Health University of Manchester Manchester UK; ^3^ Division of Informatics, Imaging, and Data Sciences University of Manchester Manchester UK

**Keywords:** CPRD, electronic health records, NHS Diabetes Prevention Programme, non‐diabetic hyperglycaemia, type 2 diabetes mellitus

## Abstract

**Aims:**

The NHS Diabetes Prevention Programme (DPP) is a behaviour‐change programme aimed at adults diagnosed with non‐diabetic hyperglycaemia (NDH), who are at higher risk of developing type 2 diabetes mellitus (Diabetes). This paper explores the heterogeneity in the effectiveness of the DPP by age, sex, BMI, and practice location deprivation (IMD).

**Methods:**

Matched cohort analysis with random‐effects parametric survival models, evaluating the association between referral to the DPP and conversion to diabetes, with interactions fitted for age, sex, BMI, and IMD.

**Results:**

18,470 patients referred to the programme were matched to 51,331 controls. None of the interactions of patient characteristics with referrals were statistically significant. For women, the difference in the HR of conversion to diabetes, compared to men, was HR = 0.94 (95% CI: 0.81, 1.08, *p* = 0.38); For those aged [18–34], HR = 0.79 (95% CI: 0.34, 1.84, *p* = 0.58) and aged [75–84] HR = 0.86 (95% CI:0.66, 1.12, *p* = 0.26) compared to those aged [55–64]. The HR for conversion was 0.88 (95% CI:0.62, 1.26, *p* = 0.49) for those with a BMI ≥ (25–29.9) kg/m^2^ and HR = 0.76 (95% CI:0.54, 1.06, *p* = 0.10) in those with a BMI ≥ 30 kg/m^2^ compared to BMI < 25 kg/m^2^. Finally, for the most deprived IMD quintile, compared to the least deprived, the difference in the conversion was HR = 1.31 (95% CI: 0.98, 1.73, *p* = 0.06).

**Conclusions:**

The DPP was effective in reducing conversion rates from NDH to diabetes as shown in our previous study results. The intervention appeared to be similarly effective by age, sex, BMI, and deprivation.

**What is already known**

- In England, the NHS Healthier You Diabetes Prevention Programme (DPP) is offered to adults with non‐diabetic hyper‐glycaemia, offering lifestyle advice to help reduce people's risk of developing type 2 diabetes.
- Our previous findings showed that individuals who were referred to the programme had a 20% lower risk of developing T2DM compared to those who were not referred to the DPP.

**What did the researchers do and find?**

- The DPP was effective in reducing conversions from NDH to DPP similarly across patient characteristics such as age, sex, weight and deprivation, and we did not observe significant effects heterogeneity across the examined strata.

**What are the implications of the study?**

- Large‐scale well‐designed public health interventions can be used to delay or prevent progression to T2DM and could be effective across different groups of patient characteristics.

## 1 INTRODUCTION

DPP is a behavioural intervention programme led by a partnership between NHS England, Public Health England and Diabetes UK. The programme is offered to adults aged 18 and over and identified as having NDH, that is, at risk of type 2 Diabetes (diabetes), to prevent or delay them from developing diabetes. In brief, individuals enrolled in the programme are offered at least 16 h of behavioural change intervention involving nutrition and exercise education, over a period of 9–12 months. Adults were eligible for the programme if they had a recent blood test showing a HBA1c within 42–47 mmol/mol or fasting plasma glucose level of 5.5–6.9 mmol/L. Participants were also invited through NHS health checks, consultation by GPs or searching practice records. Letters were then sent to participants to self‐refer.^1^

Previous analyses evaluating the programme showed that the DPP was effective in reducing conversion rates from NDH to diabetes. The results from our observational study using CPRD data showed that 6.2%; (1152 of the 1870) referred to the programme developed diabetes compared to 6.4%; (3280 of the 51,331) who were not referred to the programme.^2^

Type 2 diabetes is the most common type of diabetes, a condition when blood glucose levels are above normal, and one's body is unable to regulate it. It is a long‐term condition affecting around 7% of the UK population. In addition, it has been suggested that over a quarter of individuals with diabetes have the condition but are not aware of it.^3^

The pathogenesis of diabetes is complex and many risk factors have been linked to the development of the condition. Identifying the role and interactions of these different risk factors that play a role in the development of diabetes is important clinically as well as in understanding the progression of the disease. Risk factors for diabetes can be categorised into modifiable and non‐modifiable. Modifiable factors of diabetes could be excess weight, diet, exercise, or deprivation. Non‐modifiable risk factors of diabetes could be age, sex, ethnicity, and a family history of diabetes.^4^ Obesity has been a key factor in developing diabetes and it has been suggested that in 2012 around 62% of adults were either overweight or obese in England. It has been suggested that this increase in obesity levels will lead to a rise in diabetes cases.^5^ In addition, there is also evidence that there are sex differences in diabetes with women having a higher prevalence of impaired glucose tolerance especially in older ages while men are more likely to have NDH.^6^ There is also evidence that deprivation increased the risk of progression of NDH to diabetes,^7^ and increased risk of diabetes. Age‐related changes in the body such as a reduction in muscle mass and an increase in fat mass have been related to the increased risk of diabetes. There is also a reduction in functional cells in the islets of Langerhans leading to a reduction in insulin production in older individuals.^8^ This paper will explore whether the effectiveness of the DPP varies by age, sex, BMI, and deprivation.

## 2 METHODS

### 2.1 Data source

We used electronic health records from the Clinical Practice Research Datalink (CPRD). This is an administrative database funded by the government which provides access to anonymised NHS primary and secondary care records for public health research. This data consists of 60 million patients, of which over 16 million are currently registered patients of GP practices. The study included patients from both CPRD GOLD and Aurum databases. Data was obtained from a total of 2209 practices (GOLD:723; AURUM:1486). Data was linked to the practice‐level Index of Multiple Deprivation (IMD 2015, an area‐level aggregate across seven relevant domains^9^, ^10^) to obtain a proxy of the socioeconomic status of the patients.

### 2.2 Participants

Participants consisted of patients diagnosed with NDH (identified using Read codes) during the study period between the start of the rollout of the DPP (01st April 2016) to 31st March 2020. Read codes were used to identify patients who had NDH, and codes were also used to identify those referred to the DPP. Individuals with NDH who were referred to the DPP were considered as cases and those still identified with NDH but not having a referral code were considered controls. We selected our cohort based on a 1‐to‐1 propensity score matching approach for practice, nearest neighbour with no replacement. Our previous analysis using this cohort describes in detail the sample selection and matching process.^2^ In summary, patients were matched from referring practices to non‐referring practices over the study period, prior to matching referred individuals to non‐referred individuals. Our main outcome of interest was the interactions of covariates age, sex, BMI, and socio‐economic status in individuals who converted from NDH to diabetes.

### 2.3 Study covariates

We extracted information on the following covariates: age, sex, BMI, and IMD quintiles at baseline. We also extracted the covariates which have previously been reported^11^ to be relevant to NDH and diabetes: HbA1c, total serum cholesterol, systolic blood pressure, diastolic blood pressure, metformin, smoking status, and depression. The latest available measurement before the referral date, up until the previous 12 months, was used to define baseline total cholesterol, blood pressure, and BMI. If a value was not available, the measurement was set to missing.

Age was defined as the index date (referral date for referred to DPP and the matched control)‐date of birth. We calculated the age at referral for the matched cohort, as well as the age at the NDH diagnosis date. The age group was categorised into the following bands: 18–34, 35–44, 45–54, 55–64, 65–74, 75–84, and ≥ 85 years.

BMI values were classified into the following categories: underweight (<18.5 kg/m^2^), normal (18.5–24.9 kg/m^2^), overweight (25.0–29.9 kg/m^2^) and obese (≥30 kg/m^2^).

Total serum cholesterol in mmol/L was categorised into under 3.0, (3.0, 4.0), (4.0, 5.0), (5.0, 6.0) and 6.0 or over.

#### 2.3.1 Charlson Co‐morbidity Index

We also quantified the multi‐morbidity burden, using the Charlson Co‐morbidity Index (CCI), a widely used measure which assigns different weights to different conditions and includes: any malignancy, cerebrovascular disease, chronic pulmonary disease, congestive cardiac disease, dementia, HIV/AIDS, hemiplegia, lymphoproliferative disorders, metastatic solid tumour, mild liver disease, moderate and severe liver disease (CCI also includes diabetes with complications, which we necessarily excluded). This modified CCI was calculated using the list of validated diagnostic primary care Read codes used by Khan et al.^12^ Participants were classified as having a condition if a Read code for the condition was ever present. CCI takes integer values and was categorised as: 0, 1–2, 3–4, and >4. For CCI, we used the condition as present if it was ever recorded in the patient's primary care record. Depression was evaluated using Read codes and therapy codes which were obtained from the code lists derived from the CPRD provided on a Cambridge University repository.^13^ Participants were considered to have depression at the index date if they were recorded as depressed either by a code or if they were on relevant medication in the last 12 months. Smoking status was determined from information based on Read codes, obtained from observation and therapy files. Smoking status was recorded and categorised as “smoker”, “ex‐smoker” or “never smoked” and could vary throughout the follow‐up period. We used the last available information in the record, closest to the index date (referral date), and if there was no prior information on smoking status, we used the first mention of smoking status in the record following the index date, if any.

Prescriptions of metformin following an NDH diagnosis before diabetes diagnosis were used to dichotomise participants as ever having received metformin following their diagnosis, or not. Participants with missing data for age, sex, BMI, and deprivation (IMD) were assigned to a separate category for this variable.

### 2.4 Statistical analysis

Baseline characteristics of the cohort are presented as frequencies (%) for categorical variables, means and standard deviation (SD) for continuous data. Stata 17.0 was used for all analyses. A parametric survival model with a Weibull survival distribution and shared frailty for practice (random effects) was employed to examine associations between the covariates previously described and conversion to diabetes. In this analysis, participants were censored at the date of diagnosis of diabetes, death, or end of follow‐up, whichever happened first. Results are presented as Hazard Ratios (HR) with 95% confidence intervals. A *p*‐value of <0.05 was considered statistically significant across all analyses. We also used the margins command to interpret the median time taken for referred and non‐referred individuals in converting to diabetes, in relation to the interaction effect.^14^ Multiple imputations with chained equations (MICE) were used to impute data for variables with missing data. This method is optimal when data are missing completely at random (MCAR) or missing at random (MAR). We also tested whether data are MCAR using Little's MCAR test^15^ (MAR or missing not at random/MNAR mechanisms are untestable without external data). However, multiple imputation has been shown to provide unbiased estimates even when levels of missing data are significant, and can also offer some protection even when data are MNAR.^16^

To assess whether the effect of referral to the DPP varied across patient characteristics, we included interaction terms between referral and age, sex, BMI, and patient deprivation. We ran separate models for each of these four dimensions.

## 3 RESULTS

The final cohort after matching included 69,801 participants: 18,470 referred to DPP (cases) and 51,331 not referred to DPP (diagnosed with NDH but not referred to the programme: Controls). Baseline characteristics are presented in Appendix 1: Table A1. The mean age of patients referred to DPP was similar to those not referred. Those who were referred were more likely to be obese, with 34% of those referred to DPP and 29% of those not referred to DPP with a measurement of BMI equal to or above 30 kg/m^2^. 25% of controls and 23% of cases belonged to the lowest IMD quintile. A total of 4432 participants developed diabetes during the study period, of which 1152 (26%) were referred to DPP and 3280 (74%) were not referred to NDP.

### 3.1 Characteristics of the study cohort who developed T2D in the study period

Table 1 shows the characteristics of those individuals who converted to diabetes in the study period. The conversion to diabetes was highest in those belonging to the 55–64 age group, with 32% of cases and 35% of controls in those aged between 55 and 64 years. Individuals who developed diabetes and did not attend DPP had a higher baseline BMI than those who developed diabetes and attended NDP; however, cases were more likely to be obese, with controls having the most missing values. Conversion to diabetes increased with increasing patient deprivation, and this was similar in both groups. Developing diabetes in the study period was higher in men compared to women in both cases and controls.

**TABLE 1:** Characteristics of the cohort who developed T2DM, *N* (%) or mean (SD).^a^

	All	Cases	Controls
Type 2 diabetes	4432.0 (100.0)	1152.0 (26.0)	3280.0 (74.01)
Males	2290.0 (51.7)	603.0 (52.3)	1687.0 (51.4)
Females	2142.0 (48.3)	549.0 (47.7)	1593.0 (48.6)
Age (years)NDH diagnosis date	60.7 ± 10.8	59.7 ± 11.2	61.0 ± 10.7
*Age group (years) NDH diagnosis date*			
18–34	37.0 (0.8)	12.0 (1.0)	25.0 (0.8)
35–44	280.0 (6.3)	92.0 (8.0)	188.0 (5.7)
45–54	955.0 (21.6)	275.0 (23.9)	680.0 (20.7)
55–64	1500.0 (33.8)	368.0 (31.9)	1132.0 (34.5)
65–74	1180.0 (26.6)	297.0 (25.8)	883.0 (26.9)
75–84	444.0 (10.0)	97.0 (8.4)	347.0 (10.6)
≥85	36.0 (0.8)	11.0 (1.0)	25.0 (0.8)
BMI (kg/m^2^)	33.9 ± 7.2	33.1 ± 6.6	34.3 ± 7.4
*BMI categories*			
<18.5	6.0 (0.14)	1.0 (0.09)	5.0 (0.15)
18.5–25	185.0 (4.17)	66.0 (5.73)	119.0 (3.63)
25–29.9	721.0 (16.27)	235.0 (20.40)	486.0 (14.82)
≥30	1971.0 (44.47)	530.0 (46.01)	1441.0 (43.93)
Missing	1549.0 (34.95)	320.0 (27.78)	1229.0 (37.47)
*Patient level Deprivation Index*			
Quintile 1 (most affluent)	515.0 (11.6)	170.0 (14.8)	345.0 (10.5)
Quintile 2	750.0 (16.9)	191.0 (16.6)	559.0 (17.0)
Quintile 3	931.0 (21.0)	238.0 (20.7)	693.0 (21.1)
Quintile 4	1087.0 (24.5)	279.0 (24.2)	808.0 (24.6)
Quintile 5 (least affluent)	1149.0 (25.9)	274.0 (23.8)	875.0 (26.7)

Abbreviations: BMI, body mass index; NDH, non‐diabetic hyperglycaemia; SD, standard deviation; T2DM, type 2 diabetes mellitus.

^a^ Cases are those referred to the NHS DPP and Controls are those with NDH diagnosis in primary care, matched for sex and (within 3 years) age within 365 days of NDH diagnosis date.

### 3.2 Interaction with sex

**FIGURE 1:**
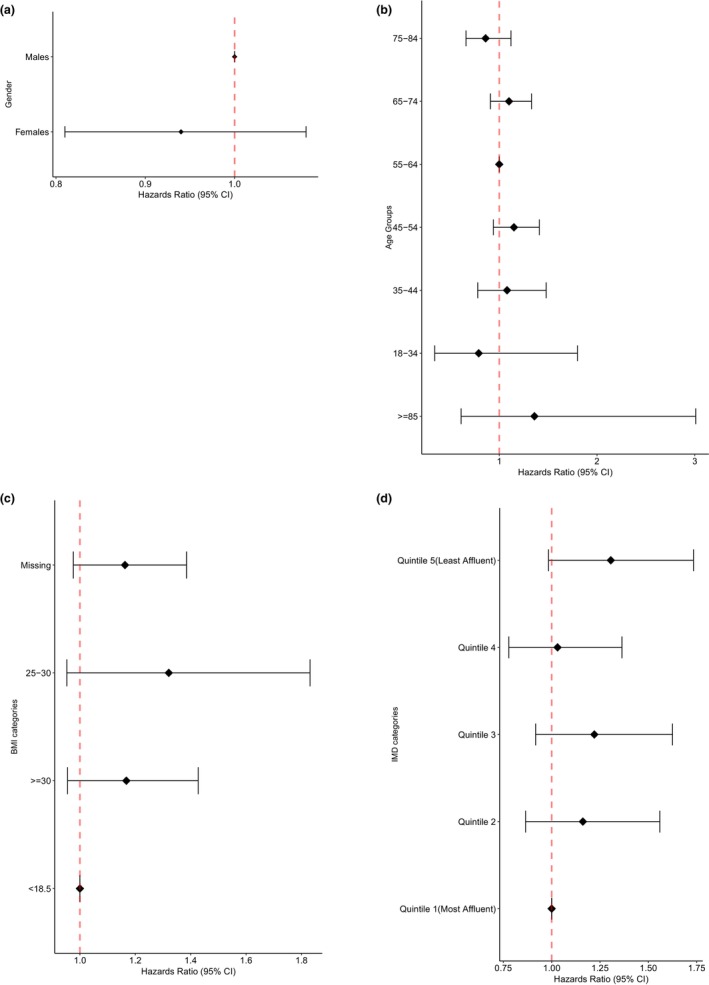
Hazard ratios of interaction for the models exploring time to conversion of referred to T2DM for patients (Cases and Controls) by baseline characteristics, with shared frailty for practice (a) sex (b) Age Groups (c) BMI categories (d) IMD categories.

The results for the survival analysis of conversion to diabetes with the interaction effect for sex are presented in Figure 1a (Appendix 2: Table A2). The results showed that overall, women were less likely to convert to diabetes with a HR of 0.80 (95% CI: 0.75, 0.86, *p* < 0.001) compared to men. When the interaction of sex was added to the model, the difference in the probability of conversion to diabetes in people who were referred to the DPP versus those who were not referred did not differ by sex [HR; 0.94, 95% CI: 0.81, 1.08, *p* = 0.38].

### 3.3 Interaction with age

Figure 1b (Appendix 2: Table A3) presents the results of the survival analysis of the participants with NDH who converted to diabetes with the interaction effect for age. The overall effect of age was compared to those aged 55–64; those who were aged 18–34, 65–74, and 75–84 were less likely to develop diabetes. The interaction effect of age on the model showed the difference in probability of conversion to diabetes between the age groups was insignificant with a *p* > 0.05.

### 3.4 Interaction with BMI

Interaction results were presented on the effect of BMI in individuals who were referred to the DPP and developed diabetes in Figure 1c (Appendix 2: Table A4). The interaction effects suggest that in those individuals who were referred to the DPP with a baseline BMI > = (25–29.9) kg/m^2^ compared to a BMI < 25 kg/m^2^, the difference in probability of conversion to diabetes was insignificant with a *p* > 0.05. Similar non‐significant results were observed in the interaction effect in those with a BMI ≥30 kg/m^2^.

### 3.5 Interaction with patient‐level deprivation (IMD quintiles)

Figure 1d (Appendix 2: Table A5) shows the model exploring results of the survival analysis of the participants with NDH who converted to diabetes with the interaction effect for IMD categories. Results of the interaction effects of IMD in participants who were referred to the DPP showed the difference in probability of conversion to diabetes in those who were in the more deprived quintiles compared to the most affluent group was insignificant with a *p* > 0.05. For example, the results showed that individuals who were in the least affluent IMD (Quintile 5) had a higher difference in probability of conversion to diabetes with a HR of 1.31 (95% CI: 0.98, 1.73) (*p* = 0.065); however, the association was insignificant with a *p* > 0.05.

## 4 DISCUSSION

We found no significant interaction between referral to the DPP and patient age, sex, BMI, and deprivation in the likelihood of developing diabetes. However, some possible insignificant trends of association were observed between increased deprivation and increased baseline BMI with an increased risk of developing diabetes, which warrants further investigation.

Results were similar to the Alive‐PD randomised controlled study, which explored an automated behavioural intervention in diabetes prevention in 340 individuals, which showed that although the intervention was effective in reducing diabetes risk and improving body weight and blood glucose levels, there were no interactions with age, sex, BMI, or ethnicity.^17^

The US Diabetes Prevention Programme, which included 3234 participants exploring lifestyle intervention, metformin intervention, and placebo, showed that participants who were in the lifestyle and metformin interventions had a lower incidence of diabetes development. They showed that the intervention was effective in all subgroups, and the effect of lifestyle intervention over metformin was larger in those who were older and had a lower BMI compared to those with increased BMI and younger.^18^ The DaQing ICT and Diabetes Study was another diabetes prevention study conducted in China, which also showed that lifestyle interventions reduced the risk of incidence of diabetes, and this was similar across BMI sub‐groups.^19^ However, these studies did not examine the role of age/sex/BMI statistically, that is, using interaction terms.

Studies exploring the interaction effects of age, sex, BMI, and deprivation on the progression of NDH to diabetes are limited. Most studies have explored the interaction effects of the variables on diabetes or NDH incidence. For example, a study conducted on Chinese adults aged over 20 years exploring the association of age and BMI on diabetes incidence showed that there was a significant association of interaction of age and BMI on the development of incidence of diabetes (age × BMI interaction, *p* < 0.001). This again did not explore the progression of diabetes from NDH, and the interaction model added both age and BMI together, whereas our study explored these variables separately.^20^ Another cross‐sectional study conducted in Shanghai showed that there was an interaction between age and risk factors of NDH and diabetes. Higher education levels and being overweight increased the risk of diabetes and NDH in those who were older (60–80 years) compared to those who were middle‐aged (40–59 years).^21^

Previous studies have shown that increased weight or obesity to be a major risk factor in the development of diabetes and is linked to the complications of the disease in both men and women.^22^ Our findings were also similar to the cross‐sectional study conducted by Bai et al. on participants aged ≥60 in China, which showed that although BMI and Waist Circumference (WC) were associated with diabetes but there was no significant interaction with the anthropometric measures and diabetes.^23^ A study conducted in a population of 27,4819 Uygur residents in China showed an interaction effect of abnormal BMI and hypertension in the development of diabetes. However, the study inclusion criteria was not prediabetes, and BMI was defined as ≥24 kg/m^2^.^24^ Younger age of diabetes diagnosis was associated with worse outcomes of diabetes such as vascular complications and mortality.^25^

Our findings are like previous studies which have shown an association of deprivation and increased risk of progression of NDH to diabetes.^26^ However interaction effects were not explored. A social experiment was conducted in the early 1990s by the US Housing Department called the “Moving to Opportunity” to study the association between socioeconomic factors and wellbeing. When individuals were followed up in 2008–2010, data showed that when followed up in 2010 showed that those who moved from an area of high levels of poverty to a lower level of poverty showed a lower prevalence of obesity and diabetes. However, no study has explored the exact mechanisms of which this change occurred.^27^

Our study has several strengths. The data was based on a large longitudinal sample which is generalizable to the UK population.^9^, ^10^ Using CPRD data we were able to access a complete medical history of the patient including other co‐morbidities and biological measures and which is broadly representative of patients' sociodemographic characteristics in terms of age and sex. Limitations of the dataset include the small number of conversions to diabetes of individuals in the different strata of age categories, sex, BMI and deprivation, which will affect the power and the precision of the estimates—hence only large effect heterogeneity would be identifiable in our analyses. Another possible limitation is that the definitions of NDH changed over time, and this might influence the categorisation of individuals as cases and controls in the study^28^ (consensus lacking 5 different definitions for prediabetes). A further possible limitation is not having anthropometric measures such as WC, a measure of visceral and ectopic fat, in our dataset, which the literature suggests is a more important risk factor in the development of diabetes.^29^

## 5 CONCLUSION

Our findings suggest that the intervention of the DPP was effective in preventing the development of diabetes in individuals diagnosed with NDH, including those in the high‐risk groups. We were not able to observe significant effects of interactions in this study.

## FUNDING INFORMATION

NIHR HS&DR. Evaluating the NHS Diabetes Prevention Programme (NHS DPP): the DIPLOMA research programme (Diabetes Prevention Long term Multimethod Assessment). The funder had no role in any aspect of this work.

## CONFLICT OF INTEREST STATEMENT

All authors have declared support from the University of Manchester for the submitted work; no financial relationships with any organisations that might have an interest in the submitted work in the previous 3 years, and no other relationships or activities that could appear to have influenced the submitted work.

## Data Availability

The data analysed in this study are subject to the following licenses/restrictions: Electronic health records are, by definition, considered "sensitive" data in the UK by the Data Protection Act and cannot be shared via public deposition because of information governance restriction in place to protect patient confidentiality. Access to data are available only once approval has been obtained through the individual constituent entity controlling access to the data. The primary care data can be requested via application to the Clinical Practice Research Datalink (www.cprd.com). Requests to access this dataset should be directed to enquiries@cprd.com.

## APPENDIX 1

**TABLE A1:** Characteristics of the matched cohort, *N* (%) or mean (SD).^a^

	All	Cases	Controls
All	69,801	18,470	51,331
Follow up from NDH diagnosis (days)	879.9 ± 804.8	980.6 ± 902.4	843.6 ± 763.4
Follow up from index date (days)	474.9 ± 311.3	482.0 ± 317.3	472.4 ± 309.1
Time from NDH diagnosis to referral date (days)	404.9 ± 741.7	498.7 ± 835.4	371.2 ± 701.9
Type 2 Diabetes (T2DM)	4432 (6.3)	1152 (6.2)	3280 (6.4)
≥1 year follow up or earlier T2DM conversion	24,39 (62.0)	6318 (60.4)	18,075 (62.7)
≥2 years follow up or earlier T2DM conversion	11,815 (30.1)	3229 (30.9)	8586 (29.8)
≥3 years follow up or earlier T2DM conversion	3111 (7.9)	919 (8.8)	2192 (7.6)
Males	33,71 (48.3)	894 (48.4)	24,77 (48.3)
Females	36,085 (51.7)	952 (51.6)	26,556 (51.7)
Age (years) NDH diagnosis date	62.4 ± 11.2	61.9 ± 11.6	62.6 ± 11.0
Age (years) referral date	63.6 ± 11.3	63.3 ± 11.8	63.7 ± 11.1
*Age group (years) NDH diagnosis date*			
18–34	513 (0.7)	191 (1.0)	322 (0.6)
35–44	3690 (5.3)	1207 (6.5)	2483 (4.8)
45–54	12,934 (18.5)	3537 (19.2)	9397 (18.3)
55–64	21,601 (31.0)	5510 (29.8)	16,091 (31.4)
65–74	20,828 (29.8)	5319 (28.8)	15,509 (30.2)
75–84	9252 (13.3)	2439 (13.2)	6813 (13.3)
≥85	983 (1.4)	267 (1.5)	716 (1.4)
BMI (kg/m^2^)	31.1 ± 6.6	30.8 ± 6.4	31.2 (6.7)
*BMI categories*			
<18.5	325 (0.47)	85 (0.46)	240 (0.47)
18.5–25	6328 (9.1)	1973 (10.7)	4355 (8.5)
25–29.9	13,731 (19.7)	4300 (23.3)	9431 (18.4)
≥30	21,355 (30.6)	6255 (33.9)	15,100 (29.4)
Missing	28,062 (40.2)	5857 (31.7)	22,205 (43.3)
*Charlson comorbidity score*			
None	38,371 (55.0)	10,362 (56.1)	28,009 (54.6)
1–2	19,109 (27.4)	4494 (24.3)	14,615 (28.5)
3–4	7168 (10.3)	1813 (9.8)	5355 (10.4)
>4	5153 (7.4)	1801 (9.8)	3352 (6.5)
*Cholesterol (%)*			
<3	1182 (1.7)	352 (1.9)	830 (1.6)
3–4	8680 (12.4)	2603 (14.1)	6077 (11.8)
4–5	15,905 (22.8)	4970 (26.9)	10,935 (21.3)
5–6	13,345 (19.1)	4441 (24.0)	8904 (17.4)
≥6	8118 (11.6)	2751 (14.9)	5367 (10.5)
Missing	22,571 (32.3)	3353 (18.2)	19,218 (37.4)
Depression	18,893 (27.1)	4275 (23.2)	14,618 (28.5)
*Smoking status*			
Current Smoker	25,751 (36.9)	9108 (49.3)	16,643 (32.4)
Ex‐smoker	30,207 (43.3)	8029 (43.5)	22,178 (43.2)
Never smoker	12,697 (18.2)	928 (5.0)	11,769 (22.9)
Missing	1146 (1.6)	405 (2.2)	741 (1.4)
Metformin	2059 (3.0)	621 (3.4)	1438 (2.8)
*Systolic blood pressure (mm Hg)*	135.4 ± 14.4	134.9 ± 14.1	135.6 ± 14.5
<120 mmHg	6824 (9.8)	2035 (11.0)	4789 (9.3)
{120‐139}mmHg	28,387 (40.7)	8414 (45.6)	19,973 (38.9)
{140–159}mmHg	18,440 (26.4)	5075 (27.5)	13,365 (26.0)
≥160 mmHg	2796 (4.0)	719 (3.9)	2077 (4.1)
Missing	13,354 (19.1)	2227 (12.1)	11,127 (21.7)
*Diastolic blood pressure (mm Hg)*	79.2 ± 9.2	79.4 ± 9.1	79.2 ± 9.2
<80 mmHg	28,178 (40.4)	8139 (44.1)	20,039 (39.0)
{80–89}mmHg	21,457 (30.7)	6168 (33.4)	15,289 (29.8)
≥90 mmHg	6812 (9.8)	1936 (10.5)	4876 (9.5)
Missing	13,354 (19.1)	2227 (12.1)	11,127 (21.7)
HBA1C (mmol/mol)	43.4 ± 3.7	43.5 ± 2.3	43.4 ± 3.9
% with HBA1C values	22,806 (32.7)	3590 (19.4)	19,216 (37.4)

^a^ Cases are those referred to the NHS DPP and Controls are those with NDH diagnosis in primary care, matched for sex and (within 3 years) age within 365 days of NDH diagnosis date.

## APPENDIX 2

**TABLE A2:** Cox proportional hazard models exploring time to conversion of referred to T2DM for patients (Cases & Controls) by baseline characteristics, with shared frailty for practice, with interaction terms added for sex.

	HR (95% CI)	*p* value
Males	Ref	
Females	0.82 (0.76–0.88)	<0.001
*Interaction terms (case#gender)*		
1#Male		
1#Female	0.94 (0.81–1.08)	0.375
*NHS DPP cohort*		
Controls		
Cases	0.82 (0.73–0.92)	0.001
*Age group (years*)		
18–34	0.51 (0.34–0.76)	0.001
35–44	0.96 (0.83–1.11)	0.595
45–54	1.08 (0.99–1.18)	0.09
55–64	Ref	
65–74	0.90 (0.83–0.98)	0.021
75–84	0.84 (0.75–0.94)	0.003
≥85	0.79 (0.56–1.11)	0.172
*Cholesterol categories (%)*		
<3	1.21 (0.97–1.52)	0.093
3–4	1.00 (0.89–1.11)	0.938
4–5	Ref	
5–6	0.98 (0.89–1.08)	0.721
≥6	1.03 (0.92–1.15)	0.581
Missing	0.85 (0.77–0.93)	0.001
*Smoking status*		
Current smoker	Ref	
Ex‐smoker	1.00 (0.93–1.08)	0.959
Never smoker	1.14 (1.03–1.25)	0.009
Missing	1.29 (1.01–1.65)	0.04
*BMI categories (kg/m* ^*2*^ *)*		
<25	Ref	
25–29.9	1.56 (1.33–1.83)	<0.001
≥30	2.38 (2.04–2.77)	<0.001
Missing	1.59 (1.36–1.86)	<0.001
Depression	1.18 (1.10–1.27)	<0.001
*CCI score*		
None	Ref	
1–2	1.14 (1.06–1.24)	0.001
3–4	1.21 (1.09–1.35)	0.001
>4	1.34 (1.18–1.53)	<0.001
Metformin	9.89 (9.01–10.86)	<0.001
*Systolic categories*		
<120 mmHg	Ref	
{120‐139} mmHg	0.82 (0.73–0.92)	0.001
{140‐159} mmHg	0.91 (0.80–1.03)	0.136
≥160	0.88 (0.72–1.07)	0.205
Missing	0.83 (0.72–0.95)	0.008
*Diastolic categories*		
<80 mmHg	Ref	
{80‐89}mmHg	1.11 (1.03–1.21)	0.01
≥90	1.15 (1.01–1.31)	0.031
Missing	—	
NDH_Ref	1.00 (1.00–1.00)	<0.001
*Patient level deprivation index*		
Quintile 1 (most affluent)		
Quintile 2	0.97 (0.85–1.12)	0.708
Quintile 3	0.92 (0.80–1.06)	0.255
Quintile 4	1.05 (0.92–1.20)	0.457
Quintile 5 (least affluent)	0.98 (0.86–1.12)	0.791

Abbreviations: BMI, body mass index; NDH, non‐diabetic hyperglycaemia; SD, standard deviation; T2DM, type 2 diabetes mellitus.

**TABLE A3:** Cox proportional hazard models exploring time to conversion of referred to T2DM for patients (Cases and Controls) by baseline characteristics, with shared frailty for practice with interaction terms added for age groups.

	HR (95% CI)	*p* value
*Age group (years)*		
18–34	0.55 (0.34–0.91)	0.021
35–44	0.94 (0.80–1.11)	0.482
45–54	1.04 (0.94–1.15)	0.447
55–64	Ref	
65–74	0.88 (0.80–0.97)	0.013
75–84	0.87 (0.77–0.99)	0.03
> = 85	0.72 (0.47–1.12)	0.143
*Interaction terms (case#agegrp)*		
1#18–34	0.79 (0.34–1.84)	0.58
1#35–44	1.08 (0.78–1.48)	0.652
1#45–54	1.15 (0.94–1.40)	0.173
1#55–64	Ref	
1#65–74	1.10 (0.90–1.33)	0.356
1#75–84	0.86 (0.66–1.12)	0.263
1# > =85	1.36 (0.60–3.06)	0.462
*NHS DPP cohort*		
Controls		
Cases	0.76 (0.66–0.88)	<0.001
Males	Ref	
Females	0.80 (0.75–0.86)	<0.001
*Cholesterol categories (%)*		
<3	1.21 (0.97–1.52)	0.096
3–4	1.00 (0.89–1.12)	0.945
4–5	Ref	
5–6	0.98 (0.89–1.07)	0.665
≥6	1.03 (0.92–1.15)	0.619
Missing	0.85 (0.77–0.93)	<0.001
*Smoking status*		
Current smoker	Ref	
Ex‐Smoker	1.00 (0.93–1.08)	0.972
Never smoker	1.14 (1.03–1.25)	0.008
missing	1.29 (1.01–1.65)	0.039
*BMI categories (kg/m* ^*2*^ *)*		
<25	Ref	
25–29.9	1.56 (1.32–1.85)	<0.001
≥30	2.38 (2.03–2.78)	<0.001
Missing	1.59 (1.35–1.87)	<0.001
Depression	1.19 (1.11–1.27)	<0.001
*CCI score*		
None	Ref	
1–2	1.14 (1.06–1.24)	0.001
3–4	1.21 (1.09–1.35)	0.001
>4	1.35 (1.18–1.53)	<0.001
Metformin	9.88 (9.03–10.82)	<0.001
*Systolic categories*		
<120 mmHg	Ref	
{120‐139}mmHg	0.82 (0.73–0.92)	0.001
{140‐159}mmHg	0.91 (0.80–1.03)	0.128
≥160	0.88 (0.73–1.06)	0.19
Missing	0.83 (0.73–0.95)	0.005
*Diastolic categories*		
<80 mmHg	Ref	
{80‐89}mmHg	1.11 (1.03–1.20)	0.009
≥90	1.15 (1.02–1.30)	0.028
Missing	—	—
NDH_Ref	1.00 (1.00–1.00)	<0.001
*Patient level deprivation index*		
Quintile 1 (Most Affluent)		
Quintile 2	0.97 (0.85–1.12)	0.719
Quintile 3	0.92 (0.80–1.05)	0.216
Quintile 4	1.05 (0.92–1.20)	0.48
Quintile 5 (least affluent)	0.98 (0.86–1.11)	0.725

Abbreviations: BMI, body mass index; NDH, non‐diabetic hyperglycaemia; SD, standard deviation; T2DM, type 2 diabetes mellitus.

**TABLE A4:** Cox proportional hazard models exploring time to conversion of referred to T2DM for patients (Cases & Controls) by baseline characteristics, with shared frailty for practice with interaction terms added for BMI categories.

	HR (95% CI)	*p* value
*BMI categories (kg/m* ^*2*^ *)*		
<25	Ref	
25–29.9	1.63 (1.33–1.99)	<0.001
≥30	2.59 (2.14–3.14)	<0.001
Missing	1.67 (1.37–2.03)	<0.001
*Interaction terms (case#BMI_categories)*		
1# < 18.5	Ref	
1#25–29.9	0.88 (0.62–1.26)	0.495
1# ≥30	0.76 (0.54–1.06)	0.104
1#Missing	0.88 (0.62–1.24)	0.466
*NHS DPP cohort*		
Controls		
Cases	0.97 (0.70–1.33)	0.833
Males	Ref	
Females	0.80 (0.75–0.86)	<0.001
*Age group (years)*		
18–34	0.51 (0.34–0.76)	0.001
35–44	0.96 (0.83–1.12)	0.623
45–54	1.08 (0.99–1.19)	0.1
55–64	Ref	
65–74	0.90 (0.83–0.98)	0.019
75–84	0.84 (0.75–0.94)	0.003
≥85	0.79 (0.55–1.13)	0.192
*Cholesterol categories (%)*		
4–5	Ref	
<3	1.22 (0.98–1.51)	0.079
3–4	1.00 (0.89–1.11)	0.93
5–6	0.98 (0.89–1.08)	0.704
≥6	1.03 (0.92–1.15)	0.6
Missing	0.85 (0.77–0.93)	0.001
*Smoking status*		
Current smoker	Ref	
Ex‐smoker	1.00 (0.93–1.07)	0.992
Never smoker	1.14 (1.03–1.25)	0.009
Missing	1.29 (1.02–1.62)	0.032
Depression	1.18 (1.11–1.27)	<0.001
*CCI score*		
None	Ref	
1–2	1.14 (1.06–1.23)	<0.001
3–4	1.21 (1.09–1.35)	0.001
>4	1.34 (1.18–1.53)	<0.001
Metformin	9.90 (9.03–10.84)	<0.001
*Systolic categories*		
<120 mmHg	Ref	
{120‐139}mmHg	0.82 (0.73–0.92)	0.001
{140‐159}mmHg	0.91 (0.80–1.03)	0.136
≥160	0.88 (0.73–1.06)	0.184
Missing	0.83 (0.73–0.95)	0.008
*Diastolic categories*		
<80 mmHg	Ref	
{80‐89}mmHg	1.11 (1.02–1.21)	0.012
≥90	1.15 (1.01–1.31)	0.032
Missing	—	—
NDH_Ref	1.00 (1.00–1.00)	<0.001
*Patient level Deprivation Index*		
Quintile 1 (most affluent)		
Quintile 2	0.97 (0.84–1.12)	0.719
Quintile 3	0.92 (0.80–1.06)	0.239
Quintile 4	1.05 (0.92–1.20)	0.47
Quintile 5 (least affluent)	0.98 (0.86–1.13)	0.812

Abbreviations: BMI, body mass index; NDH, non‐diabetic hyperglycaemia; SD, standard deviation; T2DM, type 2 diabetes mellitus.

**TABLE A5:** Cox proportional hazard models exploring time to conversion of referred to T2DM for patients (Cases & Controls) by baseline characteristics, with shared frailty for practice, with interaction terms added for quintiles of patient level deprivation index.

	HR (95% CI)	*p* value
Patient level Deprivation Index		
Quintile 1 (most affluent)		
Quintile 2	0.91 (0.77–1.07)	0.265
Quintile 3	0.84 (0.71–1.01)	0.057
Quintile 4	1.03 (0.88–1.21)	0.711
Quintile (Least Affluent)	0.88 (0.75–1.03)	0.111
Interaction effects (case#pracimd_5)		
1#Quintile 1	Ref	
1#Quintile2	1.16 (0.87–1.55)	0.314
1#Quintile3	1.22 (0.92–1.62)	0.169
1#Quintile4	1.03 (0.78–1.37)	0.835
1 #Quintile5	1.31 (0.98–1.73)	0.064
*NHS DPP cohort*		
Controls		
Cases	0.69 (0.55–0.87)	0.002
Males	Ref	
Females	0.80 (0.75–0.86)	<0.001
*Age group (years)*		
18–34	0.51 (0.34–0.76)	0.001
35–44	0.96 (0.83–1.12)	0.594
45–54	1.08 (0.99–1.18)	0.085
55–64	Ref	
65–74	0.90 (0.83–0.98)	0.016
75–84	0.84 (0.75–0.94)	0.003
> = 85	0.79 (0.56–1.11)	0.17
*Cholesterol categories (%)*		
4–5	Ref	
<3	1.22 (0.97–1.53)	0.097
3–4	1.00 (0.89–1.11)	0.949
5–6	0.98 (0.90–1.08)	0.684
≥6	1.03 (0.92–1.16)	0.611
Missing	0.85 (0.77–0.93)	0.001
*Smoking status*		
Current smoker	Ref	
Ex‐smoker	1.00 (0.93–1.08)	0.989
Never‐smoker	1.14 (1.03–1.25)	0.01
Missing	1.28 (1.01–1.64)	0.045
*BMI categories (kg/m* ^*2*^ *)*		
<25	Ref	
25–29.9	1.56 (1.33–1.83)	<0.001
≥30	2.37 (2.04–2.76)	<0.001
Missing	1.59 (1.36–1.85)	<0.001
Depression	1.19 (1.11–1.27)	<0.001
*CCI score*		
None	Ref	
1–2	1.14 (1.06–1.24)	0.001
3–4	1.21 (1.09–1.35)	0.001
>4	1.34 (1.18–1.53)	<0.001
Metformin	9.89 (9.01–10.86)	<0.001
*Systolic categories*		
<120 mmHg	Ref	
{120‐139}mmHg	0.82 (0.73–0.92)	0.001
{140‐159}mmHg	0.91 (0.80–1.03)	0.14
≥160	0.88 (0.73–1.07)	0.199
Missing	0.83 (0.73–0.94)	0.005
*Diastolic categories*		
<80 mmHg	Ref	
{80‐89}mmHg	1.11 (1.03–1.20)	0.009
≥90	1.15 (1.01–1.31)	0.031
Missing	—	—
NDH_Ref	1.00 (1.00–1.00)	<0.001

Abbreviations: BMI, body mass index; NDH, non‐diabetic hyperglycaemia; SD, standard deviation; T2DM, type 2 diabetes mellitus.
